# Single-Section Sequential MALDI-MSI Reveals Metabolic and N-Glycan Remodeling During Malignant Transformation in Hepatocellular Adenoma

**DOI:** 10.3390/metabo16040217

**Published:** 2026-03-26

**Authors:** Jianfeng Xu, Jian Sui, Da Xu, Xiaoxue Zhou, Youhong Hu, Jie Yuan, Jia Liu, Lu Lu

**Affiliations:** 1 Hepatobiliary Surgery, Department of General Surgery, Huashan Hospital, Fudan University, Shanghai 200040, China; xujianfeng@huashan.org.cn (J.X.); 10301010127@fudan.edu.cn (D.X.); 2 Cancer Metastasis Institute, Fudan University, Shanghai 200040, China; 3 Shanghai Zenith Bio Co., Ltd., Shanghai 201203, China; smp_pm@simm.ac.cn; 4 School of Pharmaceutical Science and Technology, Hangzhou Institute for Advanced Study, University of Chinese Academy of Sciences, Hangzhou 310024, China; zhouxiaoxue23@mails.ucas.ac.cn (X.Z.); yhhu@simm.ac.cn (Y.H.); 5 University of Chinese Academy of Sciences, No. 19A Yuquan Road, Beijing 100049, China; 6 Shanghai Institute of Materia Medica, Chinese Academy of Sciences, Shanghai 201203, China; yuanjie1@simm.ac.cn; 7 China-Serbia “Belt and Road” Joint Laboratory for Natural Products and Drug Discovery, Shanghai Institute of Materia Medica, Chinese Academy of Sciences, Shanghai 201203, China

**Keywords:** hepatocellular adenoma, MALDI mass spectrometry imaging, spatial metabolomics, malignant transformation

## Abstract

Background/Objectives: Malignant transformation of hepatocellular adenoma (HCA) represents a clinically significant yet incompletely understood process. Although the pathological and clinical characteristics of HCA have been extensively described, its spatial molecular heterogeneity and spatially organized molecular variation at the tissue level remain insufficiently characterized. This study aimed to establish a spatially integrated multi-omics workflow and to delineate spatially organized molecular variation across histologically defined regions from adenoma to carcinoma. Methods: A sequential dual-layer matrix-assisted laser desorption/ionization mass spectrometry imaging (MALDI-MSI) workflow was developed to acquire small-molecule metabolomic and N-glycan spatial data from the same formalin-fixed paraffin-embedded (FFPE) tissue section. Four rare HCA specimens containing focal carcinoma transformation were included in this study. Pixel-level clustering, region-based co-localization analysis, and diffusion pseudotime modeling were applied to characterize spatial metabolic and N-glycan patterns across normal liver tissue (NL), hepatocellular adenoma (HCA), and carcinoma-transformed regions within adenoma (HCA-HCC). Results: Small-molecule MSI revealed spatial metabolic stratification within HCA, with variation observed in nucleotide-related, lipid-related, sulfur-related, and sugar nucleotide–associated metabolites. Pseudotime analysis revealed a spatial ordering of samples across NL, HCA, and HCA-HCC regions, showing differences in antioxidant-associated metabolites, lipid-related features, and bile acid-related metabolites across regions. N-glycan MSI identified independent glycosylation niches, with increasing structural complexity and enrichment of highly branched glycans in carcinoma-transformed regions. Integration of metabolomic and glycomic data suggested spatially associated patterns between metabolite features and glycan structures across regions. Conclusions: This study provides spatially resolved evidence of spatially organized patterns of molecular variation across histologically defined regions of HCA. The identified metabolic and N-glycan gradients provide insights into spatial molecular organization during malignant transformation of hepatocellular adenoma.

## 1. Introduction

Hepatocellular adenoma (HCA) is a rare benign liver tumor for which epidemiological data remain limited and largely based on historical cohorts. In 1979, the incidence of HCA was reported to be 1 per million women without a history of oral contraceptive use, with a 30-fold higher prevalence among long-term oral contraceptive users [1]. Updated population-based data were not available until 2017, when the overall incidence rate of histologically verified HCA in the Danish general population was reported to be 0.07 (95% CI: 0.06–0.09) per 100,000 individuals per year [2]. Although HCAs are considered benign lesions, they may require surveillance or interventional management, including ablation or surgical resection, due to the risks of hemorrhage and malignant transformation [3]. Malignant progression to hepatocellular carcinoma (HCC) has been reported in approximately 4–10% of adenomas [4,5,6]. Among the molecular subtypes, β-catenin-activated HCAs are associated with a significantly increased risk of malignant transformation [4].

Although this subtype represents only a small proportion of the overall HCA population, its biological behavior and clinical implications differ substantially from those of benign adenomas. Once malignant transformation is established, treatment strategies and prognostic evaluation are fundamentally altered [7]. Current research on HCA has primarily focused on clinical characteristics, imaging findings, and pathological diagnostic criteria, which provide important guidance for disease identification and management [8]. However, understanding of malignant transformation in HCA remains largely confined to pathological diagnosis and clinical outcomes, while systematic characterization of its biological features at the tissue level remains limited, particularly with respect to intralesional heterogeneity and structural organization [9].

In this context, there is a pressing need for analytical approaches capable of capturing molecular information at the tissue scale to characterize the spatial heterogeneity of HCA. Mass spectrometry imaging (MSI) enables direct measurement of molecular distributions in tissue sections and allows molecular signals to be interpreted within the same spatial coordinate framework as histopathological architecture, thereby overcoming the spatial limitations inherent in conventional bulk sampling approaches [10]. Compared with molecular detection methods based on averaged signals, MSI can reveal regional molecular differences and spatial organizational patterns within lesions [11,12,13,14], providing critical technical support for delineating the intratumoral molecular phenotype of HCA. Given that biological features associated with malignant transformation may occur in a focal or spatially continuous manner, the application of MSI is particularly well suited for systematically characterizing spatial molecular phenotypes in HCA.

In the present study, we performed small-molecule and N-glycan mass spectrometry imaging on tissue sections of HCAs with focal malignant transformation and spatially co-registered these data with corresponding histopathological sections to interpret molecular distributions within their structural context. We first conducted region-specific phenotypic identification across whole tissue sections and subsequently quantified spatial relationships relative to lesion boundaries, thereby characterizing molecular gradients from normal liver parenchyma to malignant regions. Through this workflow, we aimed to explore spatially resolved molecular patterns associated with malignant transformation in HCA and to characterize region-specific metabolic and glycosylation features across histologically distinct regions.

## 2. Materials and Methods

### 2.1. FFPE Tissue Sectioning and Slide Preparation

Formalin-fixed paraffin-embedded (FFPE) HCA tissue samples were obtained from the Department of Hepatobiliary Surgery, Huashan Hospital. Informed consent for participation was obtained from all subjects involved in the study. Tissue sample analysis was approved by the Ethics Committee of Huashan Hospital, Fudan University (HIRB: 2017M-003). Tissue blocks were sectioned at a thickness of 6 μm using Leica CM1950 cryostat (Leica Biosystems, Nussloch, Germany). Sections were floated on a 40–45 °C ultrapure water bath to remove wrinkles and immediately mounted onto indium tin oxide (ITO)-coated conductive glass slides (Bruker Daltonics, Bremen, Germany). Slides were air-dried and subsequently incubated at 60 °C for 1 h to ensure firm tissue adhesion and complete paraffin softening.

### 2.2. Deparaffinization and Optical Imaging

Deparaffinization was performed by immersing the slides in freshly prepared xylene for 8 min per wash, repeated three times to ensure complete paraffin removal. Slides were then vacuum-dried for 20 min and further incubated at 60 °C for 30 min to eliminate residual solvent. High-resolution optical images of the tissue sections were acquired at 2500 dpi using OpticFilm 135i scanner (Plustek Inc., Taipei, Taiwan) prior to matrix deposition to guide region-of-interest definition during MSI acquisition.

### 2.3. Small Molecule MALDI-MSI

#### 2.3.1. Matrix Preparation and Deposition

9-Aminoacridine (9AA, Sigma-Aldrich, St. Louis, MO, USA) was prepared at 7 mg/mL in 70% acetonitrile and 30% water using LC–MS grade solvents. The matrix solution was freshly prepared and filtered through a 0.22 μm PTFE membrane before application.

Matrix deposition was carried out using HTX TM-Sprayer (HTX Technologies, Chapel Hill, NC, USA),TM Sprayer Control Software version 4.1. The sprayer was operated at a nozzle velocity of 1200 mm/min with a flow rate of 0.12 mL/min and a track spacing of 3 mm. Nitrogen was used as the nebulizing gas at 10 psi, and the spray temperature was maintained at 90 °C to promote rapid solvent evaporation and homogeneous matrix crystallization. A total of nine sequential passes were applied with a 5 s drying interval between each pass to ensure uniform coverage across the tissue surface.

#### 2.3.2. Small Molecule MSI Data Acquisition

Mass spectrometry imaging was performed on a timsTOF flex instrument (Bruker Daltonics) equipped with a 10 kHz SmartBeam 3D UV laser operating at 355 nm. Data were acquired in negative ion mode over a mass range of *m*/*z* 50–1200 at a spatial resolution of 50 μm. Each pixel was accumulated from 300 laser shots at a laser power setting of 60%. External calibration was conducted using Agilent tuning mix prior to acquisition, and calibration accuracy was verified across the mass range to ensure stable mass accuracy throughout the experiment. Regions of interest were defined using Compass flexImaging software (version 7.1, Bruker Daltonics, Bremen, Germany) based on the optical images, and acquisition parameters were controlled using timsControl software (version 4.0.5, Bruker Daltonics, Bremen, Germany).

### 2.4. Sequential In Situ N-Glycan Imaging

Following small-molecule MSI acquisition, the same tissue section was subjected to in situ enzymatic N-glycan release.

#### 2.4.1. Matrix Removal, Rehydration, and Antigen Retrieval

The previously deposited 9AA matrix was removed by immersing the slides in 70% ethanol for 7 min. Slides were sequentially rehydrated in 50% ethanol and distilled water for 2 min each, followed by equilibration in freshly prepared 10 mM ammonium bicarbonate solution for 2 min, repeated twice. To reverse formalin-induced crosslinking and enhance enzymatic accessibility, heat-induced antigen retrieval was performed by immersing the slides in 10 mM sodium citrate buffer (pH 6.0) and incubating at 90 °C for 1 h. After cooling to room temperature, slides were briefly rinsed in water and ammonium carbonate solution and subsequently vacuum-dried for 20 min.

#### 2.4.2. PNGase F Application and Incubation

Glycerol-free PNGase F (Adamas Life, 15,000 U) was diluted at a ratio of 1:14 (enzyme:H_2_O). The enzyme solution was uniformly applied using the HTX TM-Sprayer operated at a nozzle velocity of 1200 mm/min, a flow rate of 20 μL/min, and a track spacing of 3 mm, with nitrogen pressure maintained at 10 psi and the spray temperature set to 30 °C to preserve enzymatic activity.

Following deposition, the slides were incubated at 37 °C in a humidified CO_2_ incubator (0.8% CO_2_) for 12 h to enable in situ N-glycan release. Control sections processed identically but without PNGase F treatment were included to verify enzymatic specificity.

#### 2.4.3. CHCA Matrix Deposition for N-Glycan Analysis

α-Cyano-4-hydroxycinnamic acid (CHCA, Sigma-Aldrich) was prepared at 10 mg/mL in 70% acetonitrile, 30% water, and 0.1% trifluoroacetic acid. Matrix was applied using the HTX TM-Sprayer at a nozzle velocity of 1200 mm/min, a flow rate of 0.1 mL/min, and a track spacing of 3 mm, with nitrogen pressure maintained at 10 psi. The spray temperature was set to 75 °C to facilitate homogeneous crystallization. Six sequential passes were applied with a 5 s drying interval between passes.

#### 2.4.4. N-Glycan MSI Acquisition

N-glycan MSI data were acquired on the same timsTOF flex instrument in positive ion mode over a mass range of *m*/*z* 1000–4000 at a spatial resolution of 50 μm. Each pixel was generated from 300 accumulated laser shots at a laser power of 70%. External calibration using Agilent tuning mix was performed prior to acquisition.

### 2.5. Hematoxylin and Eosin (H&E) Staining

H&E-stained slides were reviewed by experienced pathologists for histopathological evaluation and region annotation (normal liver, hepatocellular adenoma, and carcinoma-transformed regions). Whole-slide images were acquired using VS200 slide scanner (Olympus Corporation, Tokyo, Japan) for downstream analysis and spatial co-registration when applicable.

### 2.6. Data Processing and Annotation

Raw MSI data were processed in SCiLS Lab (Bruker Daltonics) and exported in centroided imzML format following total ion current normalization to reduce inter-pixel variability.

The imzML datasets were uploaded to METASPACE for molecular annotation. Small-molecule identification was performed against the Human Metabolome Database (HMDB) and CoreMetabolome V3 reference libraries. N-glycan annotations were performed using a curated glycan database appropriate for PNGase F–released N-glycan structures.

Annotations were accepted based on high mass accuracy matching within instrument tolerance and false discovery rate (FDR) control as implemented by the METASPACE platform. Glycan identifications were further supported by isotopic pattern consistency and absence of signal in enzyme-negative controls.

### 2.7. Diffusion Pseudotime (DPT)

Diffusion pseudotime (DPT) was originally introduced by Haghverdi et al. [15] and later implemented in Scanpy [16]. In this study, we employed an extended implementation [17] that is capable of handling disconnected graphs. Importantly, in this study pseudotime does not represent actual chronological tumor evolution. Instead, it reflects a computational ordering of pixels based on similarity in their molecular profiles within the diffusion manifold. Therefore, the inferred trajectory should be interpreted as a spatially organized molecular gradient across tissue regions rather than a direct temporal sequence of tumor progression.

Step 1: Reuse existing PCA. The PCA embedding (X_pca, 30 components) computed during the upstream Pipeline was carried over directly, as was the UMAP embedding for visualization. Only the KNN neighbor graph was recomputed after excluding Blank pixels. Step 2: Build KNN graph (k = 50). A k-nearest-neighbor graph was constructed in PCA space. Graph connectivity was verified (required: 1 connected component). A larger k (50 vs. default 15) ensures full connectivity for the ~60 k pixel dataset. Step 3: Diffusion Map. Scanpy’s sc.tl.diffmap() computes the transition matrix of a random walk on the KNN graph, then extracts the top 10 eigenvectors (diffusion components). These capture the dominant axes of variation at multiple scales. Step 4: Root cell selection. The root cell is chosen as the Normal pixel with the most extreme value on Diffusion Component 1 (DC1), representing the point furthest from the disease boundary in diffusion space. Step 5: DPT computation.sc.tl.dpt() computes the diffusion distance from the root to every other pixel. This yields a continuous pseudotime value where nearby pixels in the diffusion manifold get similar values, naturally capturing the Normal-to-Adenoma gradient. Step 6: Direction correction. If the mean DPT of Normal > Adenoma, the axis is flipped so that pseudotime increases across regions.

## 3. Results

### 3.1. A Robust MALDI-MSI Pipeline Produced Reproducible Small-Molecule and N-Glycan Spatial Profiles from the Same FFPE Tissue Section

**Figure 1:**
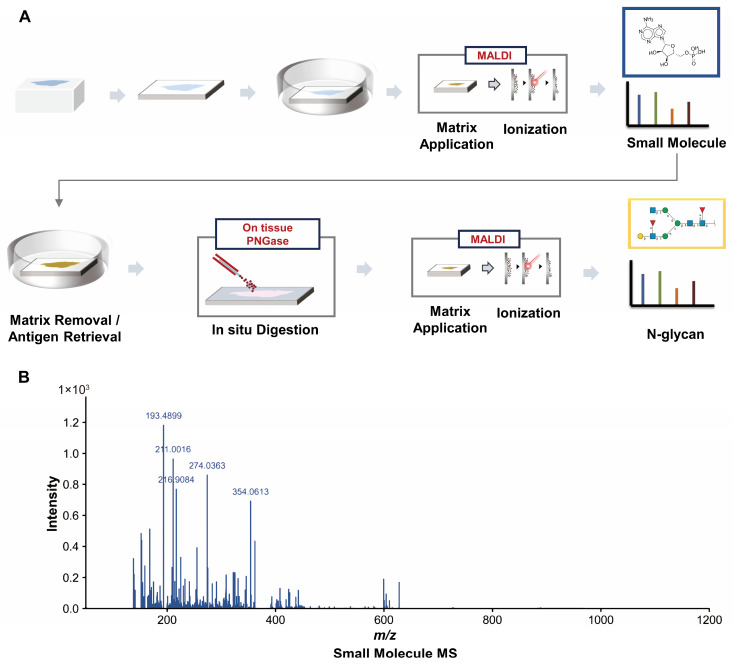

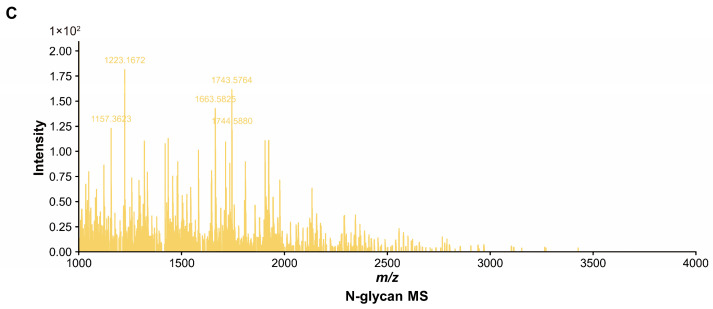
Experimental Workflow and Overview of Sequential MALDI-MSI Analysis. (**A**) Schematic illustration of the sequential dual-layer MALDI-MSI workflow performed on the same FFPE tissue section for in situ untargeted detection of small-molecule metabolites followed by N-glycans after on-tissue PNGase F digestion. (**B**) Average mass spectrum of small-molecule metabolites. (**C**) Average mass spectrum of N-glycans. The *x*-axis represents the mass-to-charge ratio (*m*/*z*), and the *y*-axis represents the mean peak intensity after root-mean-square (RMS) normalization within the corresponding *m*/*z* range.

To obtain sufficiently rich spatial molecular information from a single FFPE tissue section, we established a sequential MALDI-MSI workflow optimized for hepatocellular adenoma specimens. Small-molecule imaging was first performed, followed by on-tissue PNGase F digestion on the same section to release N-glycans, after which a second round of MSI acquisition was conducted. This approach generated spatial maps containing both small-molecule and N-glycan information from the identical tissue section (Figure 1A). By avoiding the use of adjacent sections, this workflow minimizes potential morphological variation and registration bias between sections, enabling pixel-level correspondence between molecular layers and providing a unified spatial coordinate framework for subsequent integrative analyses. Both MSI datasets underwent quality control and feature extraction within a unified analytical framework, while parameter settings were optimized according to the spectral characteristics of each molecular layer to ensure data stability and comparability. Following processing, both acquisition rounds exhibited clear spectral structures and stable signal coverage (Figure 1B,C).

For small-molecule analysis, the average whole-section spectrum showed that most signals were concentrated within the low- to mid-range *m*/*z* region, with adequate peak density and signal-to-noise performance to support spatial metabolomic profiling. Within the *m*/*z* range of 50–1200, a total of 687 features were detected, of which 60 were annotated metabolites (Figure 1B). In the subsequent N-glycan analysis, the average spectrum was primarily distributed within the *m*/*z* range of 1000–4000 and displayed multiple high-intensity, reproducible characteristic peaks consistent with the higher molecular weight of glycan structures, indicating stable and reliable signal acquisition (Figure 1C). In total, 1295 N-glycan-related features were detected, corresponding to 152 annotated glycan compositions.

Taken together, the sequential MALDI-MSI workflow enabled the acquisition of high-quality, high-coverage spatial molecular data from the same FFPE tissue section, thereby establishing a robust data foundation for downstream spatial partitioning and analysis of lesion-associated continuous molecular changes.

### 3.2. Spatial Stratification of Small-Molecule Metabolic Niches

**Figure 2:**
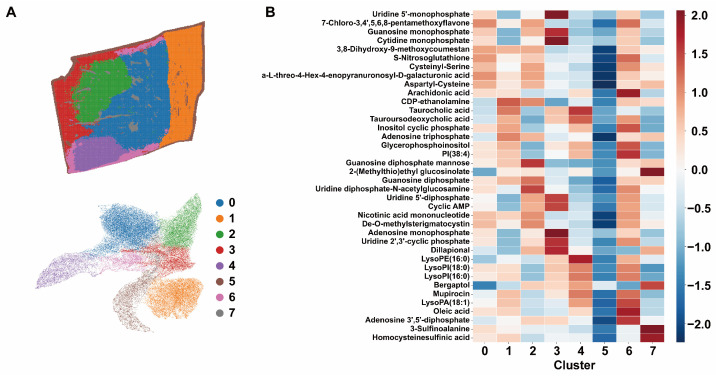

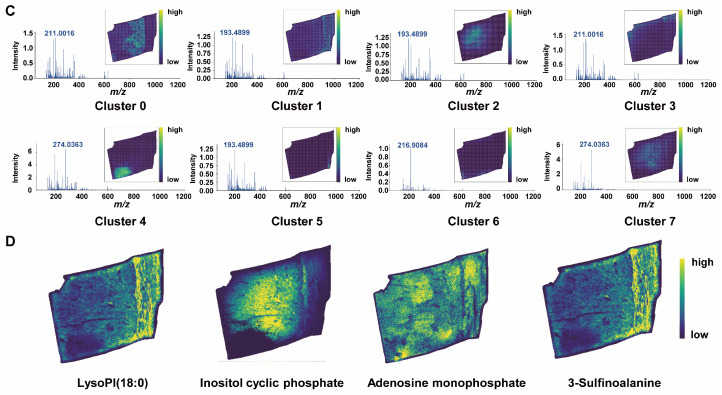
Visualization and Clustering Analysis of Small-Molecule Metabolites. All samples were subjected to UMAP dimensionality reduction and spatial clustering analysis. (**A**) Representative spatial clustering map of sample P4 and UMAP embedding of all samples. (**B**) Heatmap summarizing clustering results across all samples. The *x*-axis represents clusters, and the *y*-axis represents annotated metabolites. Color intensity indicates relative abundance. (**C**) Average mass spectra of metabolites within each cluster and ion images of the most significantly enriched metabolite. (**D**) Spatial distribution of representative metabolites in the P4 section corresponding to each cluster. Similarity scores were calculated by multiplying spatial similarity (ρ_spatial), spectral similarity (ρ_spectral), and chaos similarity (ρ_chaos). Each similarity index ranges from 0 to 1, with higher values indicating stronger similarity.

Unsupervised clustering of pixel-level small-molecule metabolic profiles stably partitioned the hepatocellular adenoma tissue section into eight distinct metabolic niches (clusters 0–7), which in this study refer to spatially localized tissue regions exhibiting distinct metabolite abundance patterns. The spatial distribution of clusters was interpreted in conjunction with H&E-stained images; clusters 2, 3, and 4 were predominantly localized in carcinoma-associated regions; clusters 0, 6, and 7 in hepatocellular adenoma areas; and cluster 1 in adjacent normal liver tissue (Figure 2A, Figure S1A–C). Each cluster was characterized by a relatively concentrated set of discriminative metabolites, forming cluster-specific molecular fingerprints at the metabolite level (Figure 2B).

At the level of nucleotides and their phosphorylated derivatives, cluster 3 exhibited the most pronounced global elevation. Uridine 5′-monophosphate (UMP), cytidine monophosphate (CMP), guanosine monophosphate (GMP), adenosine monophosphate (AMP), adenosine triphosphate (ATP), and cyclic AMP (cAMP) were consistently enriched in this niche, with cluster 3 showing higher levels of nucleotide-related metabolites. In contrast, cluster 5 showed a consistent reduction across nearly all nucleotide-related metabolites, along with decreased levels of inositol cyclic phosphate and several lipid-associated signals, reflecting a distinct pattern of lower metabolite abundance.

At the lipid level, cluster 6 showed the most prominent enrichment pattern. LysoPI(18:0), LysoPI(16:0), LysoPA(18:1), PI(38:4), and oleic acid were globally elevated within this niche, indicating a distinct lysophospholipid- and fatty acid-associated metabolic signature. Notably, nucleotide-related molecules such as adenosine 3′,5′-diphosphate were also concurrently increased, suggesting an association with lipid-related and nucleotide-related metabolites within this region.

Cluster 7 was characterized by enrichment of sulfur-related metabolites, including homocysteinesulfinic acid, 3-sulfinolalanine, and 2-(methylthio)ethyl glucosinolate, defining a niche with a distinct sulfur metabolism signature. In contrast, cluster 2 was enriched in sugar nucleotides and glycan precursors, such as guanosine diphosphate mannose (GDP-mannose) and uridine diphosphate N-acetylglucosamine (UDP-GlcNAc), forming a “sugar nucleotide–high” profile distinct from the nucleotide-enriched cluster 3. Cluster 4 showed relatively elevated levels of inositol cyclic phosphate and glycerophosphoinositol, suggesting association with inositol phosphate-related metabolites (Figure 2B). These metabolite-defined niches were consistently reflected at the spectral level as reproducible differences in peak group composition (Figure 2C and Figure S1D–F). Nucleotide-enriched niches displayed prominent features in the low- to mid-*m*/*z* range, whereas lipid-enriched niches showed stronger signals within *m*/*z* ranges corresponding to lysophospholipids and fatty acids. Ion imaging of representative metabolites, including LysoPI(18:0) and inositol cyclic phosphate, demonstrated strong spatial concordance between molecular distributions and niche boundaries (Figure 2D and Figure S1G).

### 3.3. Spatial Stratification of N-Glycan Signatures Reveals Distinct Glycosylation Niches

**Figure 3:**
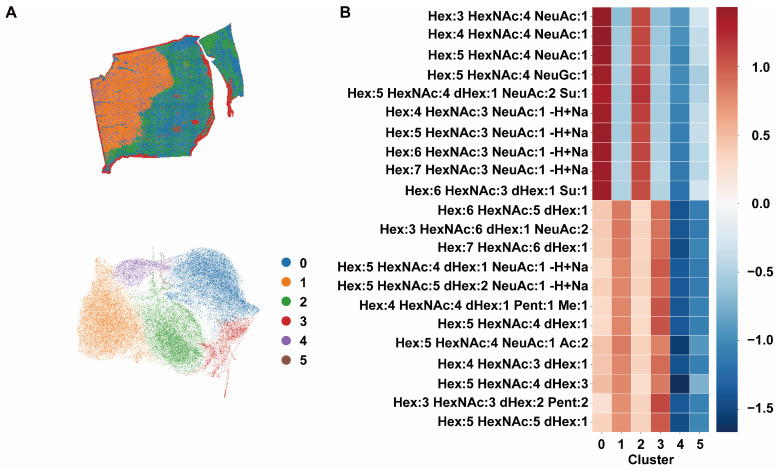

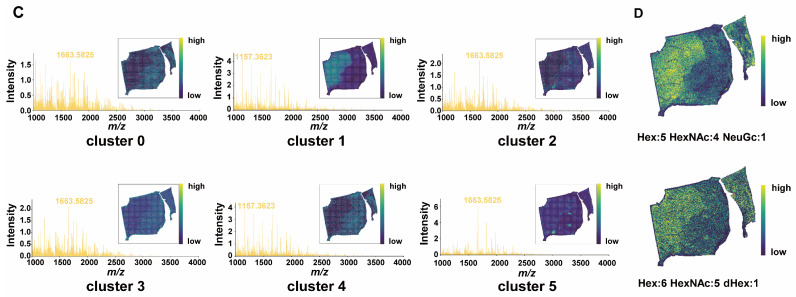
Visualization and Clustering Analysis of N-Glycan MSI Data. All samples were subjected to UMAP dimensionality reduction and spatial clustering analysis. (**A**) Representative spatial clustering map of sample P4 and UMAP embedding of all samples. (**B**) Heatmap summarizing N-glycan clustering results across all samples. The *x*-axis represents clusters, and the *y*-axis represents annotated N-glycan compositions. Color intensity indicates relative abundance. (**C**) Average mass spectra of N-glycans within each cluster and ion images of the most significantly enriched N-glycan species. (**D**) Spatial distribution of representative N-glycans in the P4 section corresponding to each cluster. Similarity scores were calculated as the product of spatial similarity (ρ_spatial), spectral similarity (ρ_spectral), and chaos similarity (ρ_chaos), with values ranging from 0 to 1.

Following small-molecule imaging, on-tissue PNGase F digestion was performed on the same FFPE section, and N-glycan MSI data were acquired. Unsupervised clustering of pixel-level N-glycan spectra partitioned the tissue section into six glycan niches (clusters 0–5) (Figure 3A and Figure S2A–C). Each niche was characterized by a relatively concentrated set of glycan composition features, forming structurally distinguishable glycosylation fingerprints (Figure 3B). At the global level, differences among glycan niches were primarily reflected in the degree of sialylation, the HexNAc/Hex compositional ratio, and the presence of dHex (fucose) modifications. Clusters 0 and 2 exhibited more prominent sialylated complex-type N-glycan features, with structures such as Hex:3–5 HexNAc:4 NeuAc:1 and related compositions consistently enriched in these niches. In contrast, clusters 4 and 5 showed overall reduced levels of multiple sialylated glycans and displayed distinct combinatorial patterns involving dHex-containing structures or higher HexNAc content, forming glycosylation fingerprints distinct from clusters 0 and 2. Cluster 3 demonstrated relative enrichment of several high-HexNAc, high-complexity structures (e.g., Hex:6 HexNAc:5 dHex:1), suggesting accumulation of more highly branched N-glycan compositions within this niche. Cluster 1 exhibited comparatively elevated signals for a subset of glycans containing additional structural modifications, generating a structurally independent compositional profile (Figure 3B). These compositional differences were consistently reflected at the spectral level as reproducible variations in peak cluster patterns (Figure 3C and Figure S2D–F). Ion imaging of representative glycan structures further demonstrated strong spatial concordance between their molecular distributions and the corresponding glycan niche boundaries (Figure 3D and Figure S2G).

### 3.4. Spatial Variation in Metabolic Features Across Histologically Defined Regions

**Figure 4:**
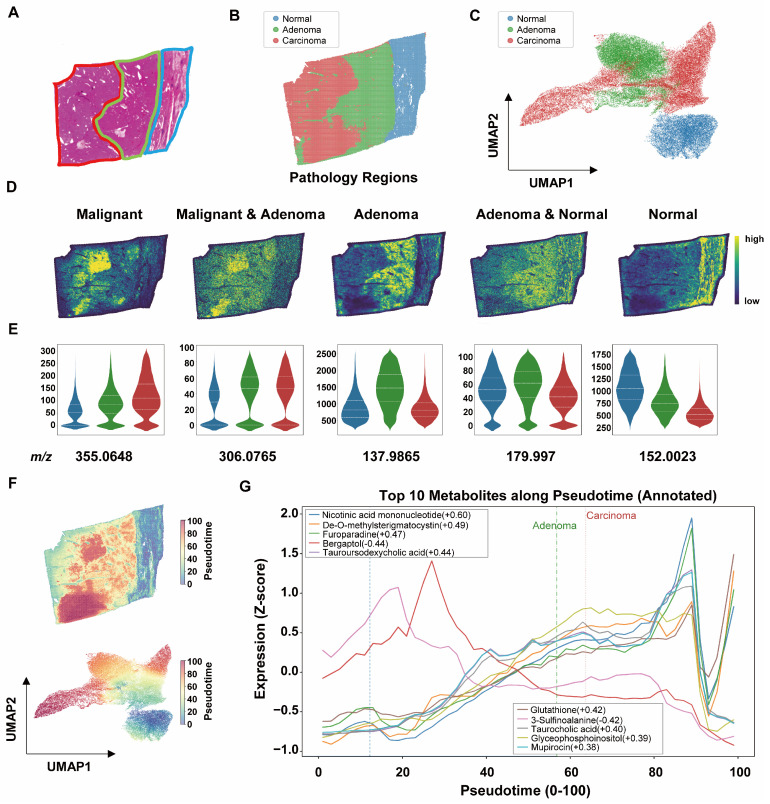
Region-Based Co-Localization Analysis of Small-Molecule Metabolites. (**A**) Histopathological annotation of representative sample P4. Blue indicates NL, green indicates HCA, and red indicates carcinoma-transformed regions. (**B**) In situ visualization of annotated regions in the P4 section. (**C**) UMAP embedding of all samples after region assignment. (**D**) Spatial visualization of representative metabolites across different pathological regions in P4. (**E**) Violin plots showing statistical comparison of metabolite abundance among NL, HCA, and HCA-HCC regions. The *y*-axis represents normalized abundance. (**F**) Spatial diffusion pseudotime analysis (ordering across NL, HCA, and HCA-HCC regions) visualized in situ in P4 and in UMAP space for all samples. (**G**) Top 10 metabolites varying along pseudotime. The *x*-axis (0–100) represents pseudotime ordering across regions, and the *y*-axis represents z-score normalized metabolite abundance.

Based on small-molecule spatial distribution data obtained by MALDI-MSI, pixel-level spectra were stratified according to histopathological regions (Figure 4A and Figure S3A–C) into normal liver tissue (NL), hepatocellular adenoma (HCA), and carcinoma-transformed regions within adenoma (HCA-HCC) (Figure 4B,C). Region-specific characteristic metabolites were identified and demonstrated strong discriminatory capacity across the three pathological compartments. Representative metabolite ion images (Figure 4D and Figure S3G) and corresponding violin plot quantifications (Figure 4E) illustrate their distinct regional distributions.

To characterize spatial molecular variation, diffusion pseudotime analysis was used to generate a computational ordering of pixels across histologically defined regions based on molecular similarity (Figure 4D,E and Figure S3G). The three tissue categories were distributed along the principal axis of the diffusion space, indicating a continuous pattern of molecular variation across regions (Figure 4F and Figure S3D–F). Across the pseudotime ordering, molecular features showed gradual variation in their spatial distributions. Redox-associated metabolites, including glutathione, showed increasing trends across the pseudotime ordering of pixels. In contrast, sulfur-related metabolites such as sulfinoalanine declined progressively, showing opposite trends across regions, with glutathione increasing and sulfur-related metabolites decreasing (Figure 4G).

Consistently, the oxidation-associated metabolite bergaptol showed a decreasing trend across the pseudotime ordering. In terms of lipid and membrane metabolism, glycerophosphoinositol exhibited an increasing trend along the trajectory, which may be associated with changes in phosphatidylinositol-related membrane processes. Regarding bile acid metabolism, taurine-conjugated bile acids, including tauroursodeoxycholic acid (TUDCA) and taurocholic acid (TCA), were relatively more abundant in carcinoma transformed regions and showed an upward trend across regions. These observations may reflect region-associated differences in bile acid metabolism; however, given the exploratory nature of this study, these patterns should be interpreted cautiously and require further validation (Figure 4G). Collectively, these observations suggest spatially organized patterns of molecular variation across histologically defined regions.

### 3.5. Spatial Variation in N-Glycan Features Across Histologically Defined Regions

**Figure 5:**
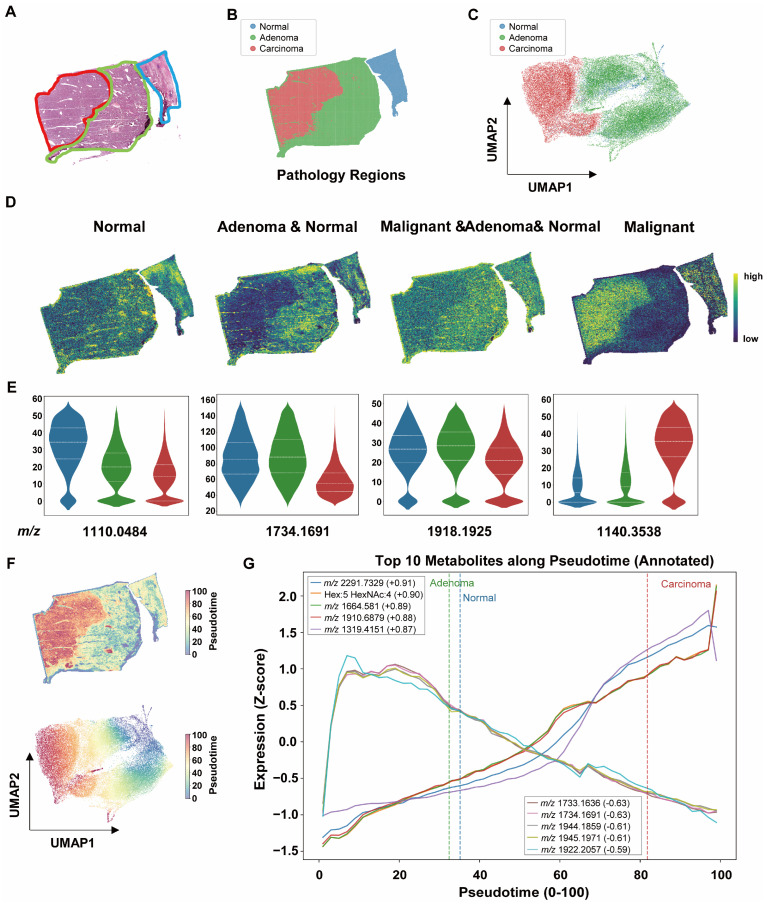
Region-Based Co-Localization Analysis of N-Glycans. (**A**) Histopathological annotation of representative sample P4. Blue indicates normal liver tissue, green indicates HCA, and red indicates carcinoma-transformed regions within adenoma (HCA-HCC). (**B**) In situ visualization of annotated regions in the P4 section. (**C**) UMAP embedding of all samples after region assignment. (**D**) Spatial visualization of representative N-glycans across different pathological regions in P4. (**E**) Violin plots showing statistical comparison of N-glycan abundance among NL, HCA, and HCA-HCC regions. The *y*-axis represents normalized abundance. (**F**) Spatial diffusion pseudotime analysis (ordering across NL, HCA, and HCA-HCC regions) visualized in situ in P4 and in UMAP space for all samples. (**G**) Top 10 N-glycans varying along pseudotime. The *x*-axis (0–100) represents pseudotime ordering across regions, and the *y*-axis represents z-score normalized N-glycan abundance.

Based on N-glycan spatial distribution data obtained by MALDI-MSI, pixel-level spectra were stratified according to histopathological regions (Figure 5A and Figure S4A–C) into normal liver tissue (NL), hepatocellular adenoma (HCA), and carcinoma-transformed regions within adenoma (HCA-HCC) (Figure 5B,C). Region-specific N-glycans were characterized, and these features effectively discriminated among the three pathological compartments. Representative ion images of characteristic N-glycans (Figure 5D and Figure S4G) and corresponding violin plot quantifications (Figure 5E) demonstrate their distinct regional distributions.

Diffusion pseudotime analysis was subsequently applied to generate a computational ordering of glycan features across histologically defined regions. The ordering revealed that the three tissue categories were distributed along the principal axis, indicating a gradual variation pattern of N-glycan features across regions (Figure 5F and Figure S4D–F). Along the pseudotemporal axis, N-glycan structural complexity increased progressively. Highly branched and multi-antennary N-glycans were enriched in carcinoma-transformed regions compared with adenoma regions.

Dynamic trend analysis grouped differentially expressed N-glycans into distinct variation patterns. One module exhibited an increasing pattern across the spatial ordering, with representative structures including Hex:5 HexNAc:4. In contrast, another module showed a gradual decline, with the top five decreasing N-glycans corresponding to *m*/*z* 1733.1636, 1734.1691, 1944.1859, 1945.1971, and 1922.2057 (Figure 5G).

Spatial visualization further confirmed that N-glycans with increased structural complexity and higher levels of modification were preferentially enriched in carcinoma-transformed regions, indicating differences in glycan complexity across histologically defined regions.

Collectively, these findings indicate spatially organized variation in N-glycan features across histologically defined regions. Compared with normal liver and adenoma regions, carcinoma-transformed areas showed enrichment of more highly branched and complex N-glycan structures. These patterns suggest that N-glycan structural complexity is associated with histological differences observed in hepatocellular adenoma.

## 4. Discussion

In this study, we established a sequential MALDI-MSI workflow that enables the acquisition of both small-molecule and N-glycan spatial distributions from the same FFPE tissue section, thereby achieving pixel-level alignment of metabolomic and glycomic data. Compared with conventional multi-omics integration strategies that rely on adjacent serial sections, this approach minimizes morphological variation and registration bias between sections, providing a more reliable technical foundation for cross-molecular spatial integration [18]. In liver tumors, which are characterized by marked structural heterogeneity and regionally variable pathological features [19], maintaining spatial consistency is particularly critical for accurately resolving spatial molecular variation across regions [20]. Our results demonstrate that this dual-layer MSI workflow achieves stable signal acquisition and broad molecular coverage, supporting its applicability to spatial metabolomic and glycomic profiling in complex tissues.

FFPE tissues represent an important resource for clinical studies because they are often associated with detailed pathological evaluation and long-term clinical follow-up information. However, FFPE processing and sequential MSI acquisition may introduce potential perturbations, including chemical alterations and molecular delocalization. To minimize these effects, small-molecule MSI was performed prior to any antigen retrieval or de-crosslinking procedures, and subsequent on-tissue enzymatic digestion for N-glycan imaging was conducted using a low-solvent spraying strategy to reduce analyte diffusion. This study demonstrates the feasibility of applying a sequential MSI workflow to obtain complementary molecular information from the same FFPE tissue section. However, potential interference with spatial information cannot be fully excluded. Accordingly, the results are interpreted with caution and presented in a more descriptive manner. Further optimization based on this workflow may improve spatial fidelity, highlighting its potential value for integrated spatial analyses.

Small-molecule MSI revealed clear and stable metabolic stratification within hepatocellular adenoma tissue rather than a metabolically homogeneous landscape, consistent with previous spatial metabolomics studies in liver tumors [21,22]. Distinct spatial patterns of metabolite features were observed across regions, including nucleotide-related, lipid-related, sugar nucleotide-associated, and sulfur-related metabolites, indicating spatial molecular heterogeneity within the tissue. Regions with relatively higher levels of nucleotide-related metabolites were observed, as reported in previous studies [23,24]. Areas enriched in lipid-related and lysophospholipid-associated metabolites were also identified, consistent with prior observations [25,26]. In addition, spatial variation in sulfur-related metabolites was observed across regions, in line with previous reports [27]. A relatively small proportion of detected *m*/*z* features could be confidently annotated as metabolites, which limits direct biochemical interpretation. However, unannotated MSI features may still capture biologically meaningful spatial information and have been widely used in spatial metabolomics analyses [28]. Together, these observations provide a spatial overview of metabolite feature variation within hepatocellular adenoma. The spatial metabolic patterns identified in this study may warrant further investigation in future studies. In particular, differences in antioxidant-associated metabolites and lipid-related features were observed across regions. Although these observations remain exploratory, they may provide a basis for future studies aimed at further characterizing region-specific molecular features in hepatocellular adenoma.

Diffusion pseudotime analysis revealed a continuous ordering of pixels based on molecular similarity across histologically defined regions. Along this path, glutathione levels increased, while sulfur-related metabolites declined across regions. Similar patterns have been reported in previous studies [29]. Concurrently, phospholipid-associated metabolites and taurine-conjugated bile acids were more abundant in carcinoma-associated regions. Comparable observations have also been described in prior studies [25,30]. These coordinated patterns suggest spatially structured molecular variation across tissue regions.

Glycosylation is a critical post-translational modification whose dysregulation has been implicated in numerous diseases [31,32]. MALDI-MSI offers substantial potential for spatial characterization of N-glycan expression in heterogeneous tissues [33] and can achieve enhanced detection sensitivity through methodological and instrumental advances [34,35,36]. At the glycosylation level, N-glycan MSI revealed an independent and organized spatial stratification pattern. Across regions, N-glycan structures displayed increasing complexity, with multi-branched and highly antennary glycans significantly enriched in carcinoma regions, whereas simpler structures declined. Highly branched N-glycans have been shown to enhance receptor stability and amplify signaling pathways [37], which has been reported to be associated with tumor-related signaling features and intercellular interaction patterns. Notably, increased levels of sugar nucleotide precursors at the small-molecule level provide a potential substrate basis for glycan structural complexity, supporting functional coupling between metabolic reprogramming and glycosylation remodeling [38,39]. Alterations in metabolic state may therefore influence not only energy and biosynthetic demands but also reshape protein glycosylation patterns through regulation of glycosyl donor availability.

Collectively, our observations indicate spatially associated patterns between metabolite features and glycan structures across histologically defined regions. These cross-layer observations suggest that adenoma and carcinoma regions exhibit coordinated differences across multiple molecular networks, although further validation is required.

This study has several limitations. One limitation relates to metabolite identification in MALDI-MSI datasets. In this study, metabolite annotations were primarily obtained through accurate mass matching using the METASPACE platform with false discovery rate (FDR) control. Although this approach provides high-confidence putative annotations for spatial metabolomics studies, MSI-based metabolite identification without complementary MS/MS validation cannot unambiguously confirm molecular identity. Therefore, the reported metabolites should be considered tentative annotations rather than definitive structural identifications. Consequently, the biochemical interpretations presented in this study should be viewed cautiously and mainly as hypothesis-generating observations. Future studies integrating targeted MS/MS validation or orthogonal metabolomics approaches will be necessary to further confirm metabolite identities and refine pathway-level interpretations. The current analysis is based on spatial associations and does not establish direct causal relationships. N-glycan structural characterization was limited to compositional annotation without detailed linkage or site-specific information. Additionally, due to the rarity of adenoma specimens containing focal carcinoma transformation, the sample size was limited. Future studies with expanded cohorts across additional tumor types, combined with metabolic flux analysis, glycosyltransferase expression profiling, and functional perturbation models, will help further elucidate the mechanistic basis of coordinated metabolic and glycosylation remodeling during malignant transformation.

In summary, through spatial integration of metabolomic and glycomic data, this study revealed spatially organized patterns of molecular variation across histologically defined regions in hepatocellular adenoma. These findings provide exploratory, spatially resolved observations of metabolomic and glycomic differences among tissue regions and may offer a basis for future studies aimed at further validating their biological and clinical relevance.

## 5. Conclusions

In this study, we established a sequential dual-layer MALDI-MSI spatial integration workflow that enables pixel-level alignment of small-molecule metabolomic and N-glycan data within the same FFPE tissue section. This approach provides a robust multi-omics spatial analysis platform for structurally heterogeneous tumors such as hepatocellular adenoma. By improving spatial precision and eliminating inter-section registration bias, this method facilitates refined delineation of potentially malignant subregions within complex lesions.

Using this spatial integration strategy, we observed spatially organized patterns of molecular variation across histologically defined regions from hepatocellular adenoma to carcinoma. Differences in antioxidant-associated metabolites, membrane lipid-related features, bile acid-related metabolites, and N-glycan structures were observed in carcinoma-associated regions. These findings suggest that molecular features vary across regions and may follow gradual spatial patterns, rather than representing abrupt changes during malignant transformation. Such spatially resolved molecular patterns may provide a basis for future studies aimed at characterizing region-specific alterations within adenomatous lesions.

From a clinical perspective, the potential relevance of these spatial molecular patterns remains to be further investigated. The spatial variation observed in metabolism and glycosylation may provide preliminary insights into region-specific molecular features; however, their applicability for assessing malignant potential or guiding clinical decision-making requires further validation in larger cohorts. Moreover, metabolic antioxidant-associated features and glycosylation-related patterns may warrant further investigation in future studies.

Overall, by integrating spatial metabolomic and glycomic data, this study provides spatially resolved observations of molecular variation in hepatocellular adenoma and offers a basis for future studies aimed at further evaluating their biological and clinical relevance.

## Data Availability

The data that support the findings of this study are available from the corresponding authors J.Y., J.L. and L.L. upon reasonable request due to ethical or privacy restrictions.

## Supplementary Materials

The following supporting information can be downloaded at https://www.mdpi.com/article/10.3390/metabo16040217/s1, Figure S1: Extended data supporting Figure 2; Figure S2: Extended data supporting Figure 3; Figure S3: Extended data supporting Figure 4; Figure S4: Extended data supporting Figure 5.

## Abbreviations

The following abbreviations are used in this manuscript:

HCA	Hepatocellular adenoma
FFPE	Formalin-fixed paraffin-embedded
MSI	Mass spectrometry imaging
MALDI	Matrix-assisted laser desorption/ionization
HMDB	Human Metabolome Database
FDR	False discovery rate
DPT	Diffusion Pseudotime
PCA	Principal Component Analysis
PI	Phosphatidylinositol
PA	Phosphatidic acid
NL	Normal Liver
