# Supplementary material for: Single-Section Sequential MALDI-MSI Reveals Metabolic and N-Glycan Remodeling During Malignant Transformation in Hepatocellular Adenoma
Source: Metabolites. 2026 Mar 26;16(4):217. doi: 10.3390/metabo16040217 (PMC13117799; doi:10.3390/metabo16040217)
Supplement: Supplementary file 1 [file metabolites-16-00217-s001.zip › Supplement Figure Legend.pdf]

Figure S1. Extended data supporting Fig. 2.
(A–C) Spatial segmentation of P1–P3 samples.
(D–F) Ion images of the most enriched metabolites across clusters.
(G) Spatial characteristics of the representative metabolites.

Figure S2. Extended data supporting Fig. 3.
(A–C) Spatial segmentation of P1–P3 samples.
(D–F) Ion images of the most enriched metabolites across clusters.
(G) Spatial characteristics of the representative metabolites.

Figure S3. Extended data supporting Fig. 4.
(A–C) Pathological region annotation of P1–P3 samples (Blue represents normal liver parenchyma, green represents adenomatous tissue, and red represents carcinoma arising within adenoma.).
(D–F). Spatial pseudotime trajectory of small-molecule metabolites in P1–P3 samples (Normal → Adenoma → Carcinoma).
(G) Spatial ion images of small-molecule metabolites enriched in distinct pathological regions.

Figure S4. Extended data supporting Fig. 5.
(A–C) Pathological region annotation of P1–P3 samples (Blue represents normal liver parenchyma, green represents adenomatous tissue, and red represents carcinoma arising within adenoma.).
(D–F). Spatial pseudotime trajectory of N-glycan metabolites in P1–P3 samples (Normal → Adenoma → Carcinoma).
(G) Spatial ion images of N-glycan metabolites enriched in distinct pathological regions.
