# Supplementary figures and images for: Single-Section Sequential MALDI-MSI Reveals Metabolic and N-Glycan Remodeling During Malignant Transformation in Hepatocellular Adenoma
Source: Metabolites. 2026 Mar 26;16(4):217. doi: 10.3390/metabo16040217 (PMC13117799; doi:10.3390/metabo16040217)

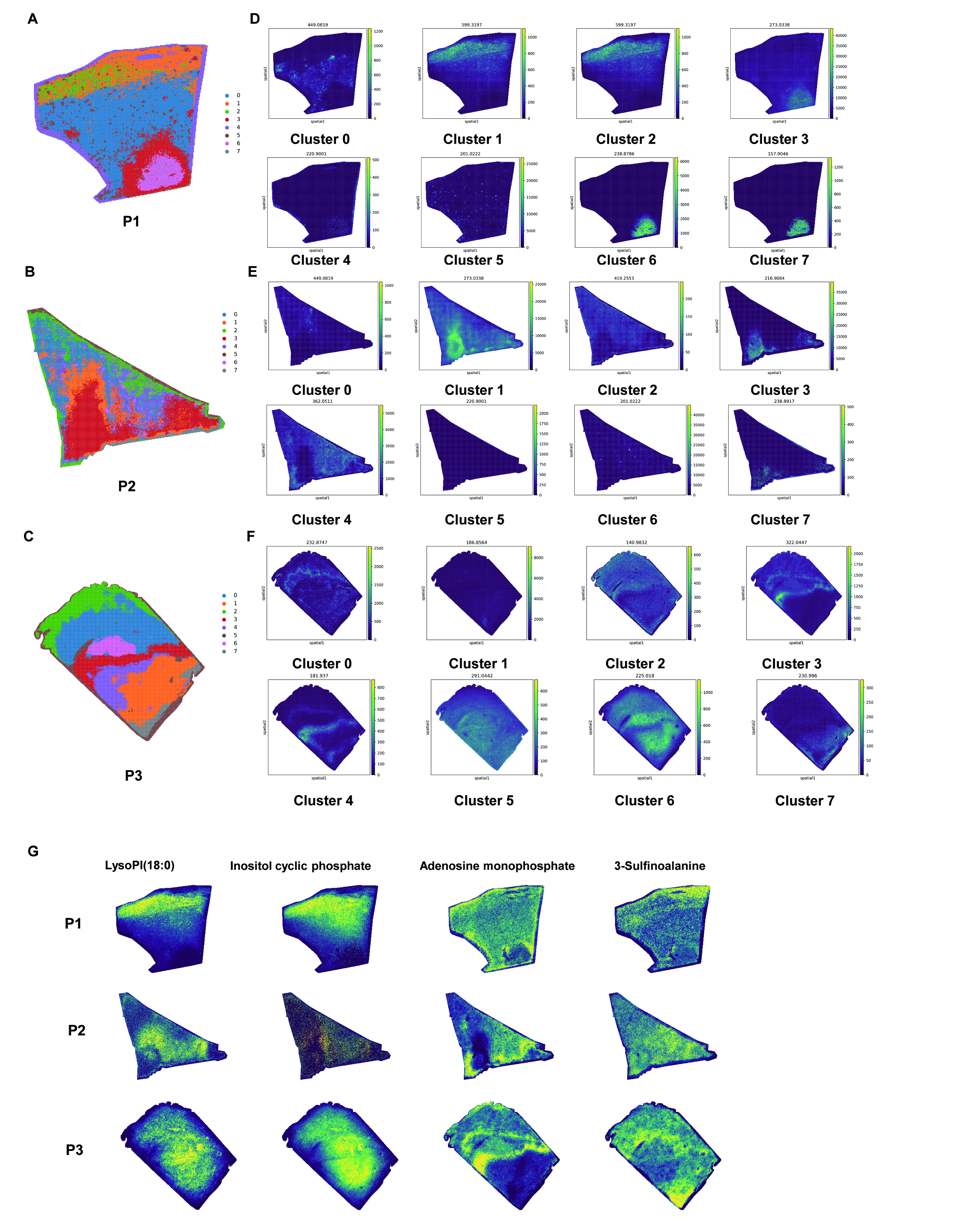

Supplement: Supplementary file 1 [file metabolites-16-00217-s001.zip › Figure S1.tif]

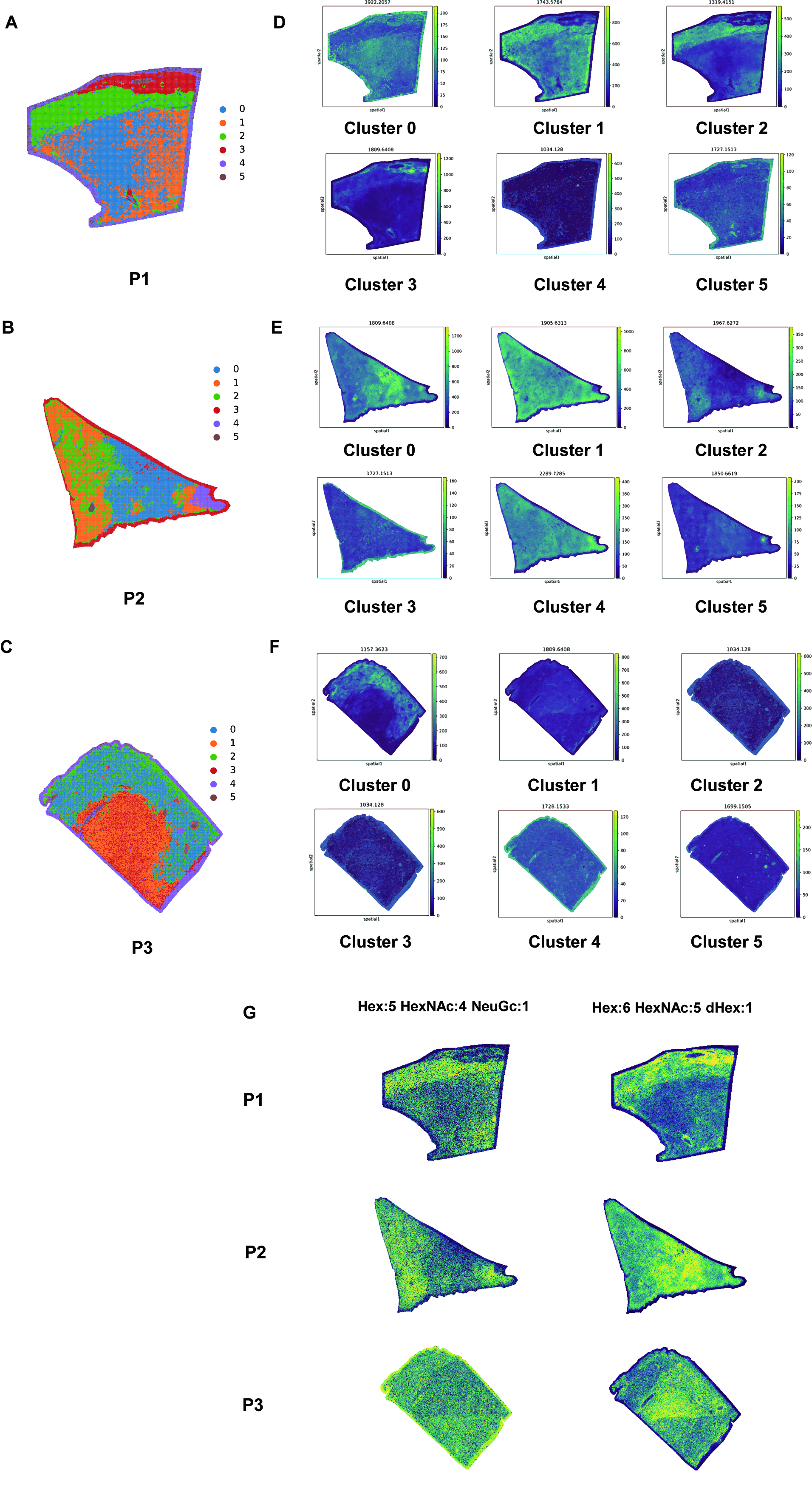

Supplement: Supplementary file 1 [file metabolites-16-00217-s001.zip › Figure S2.tif]

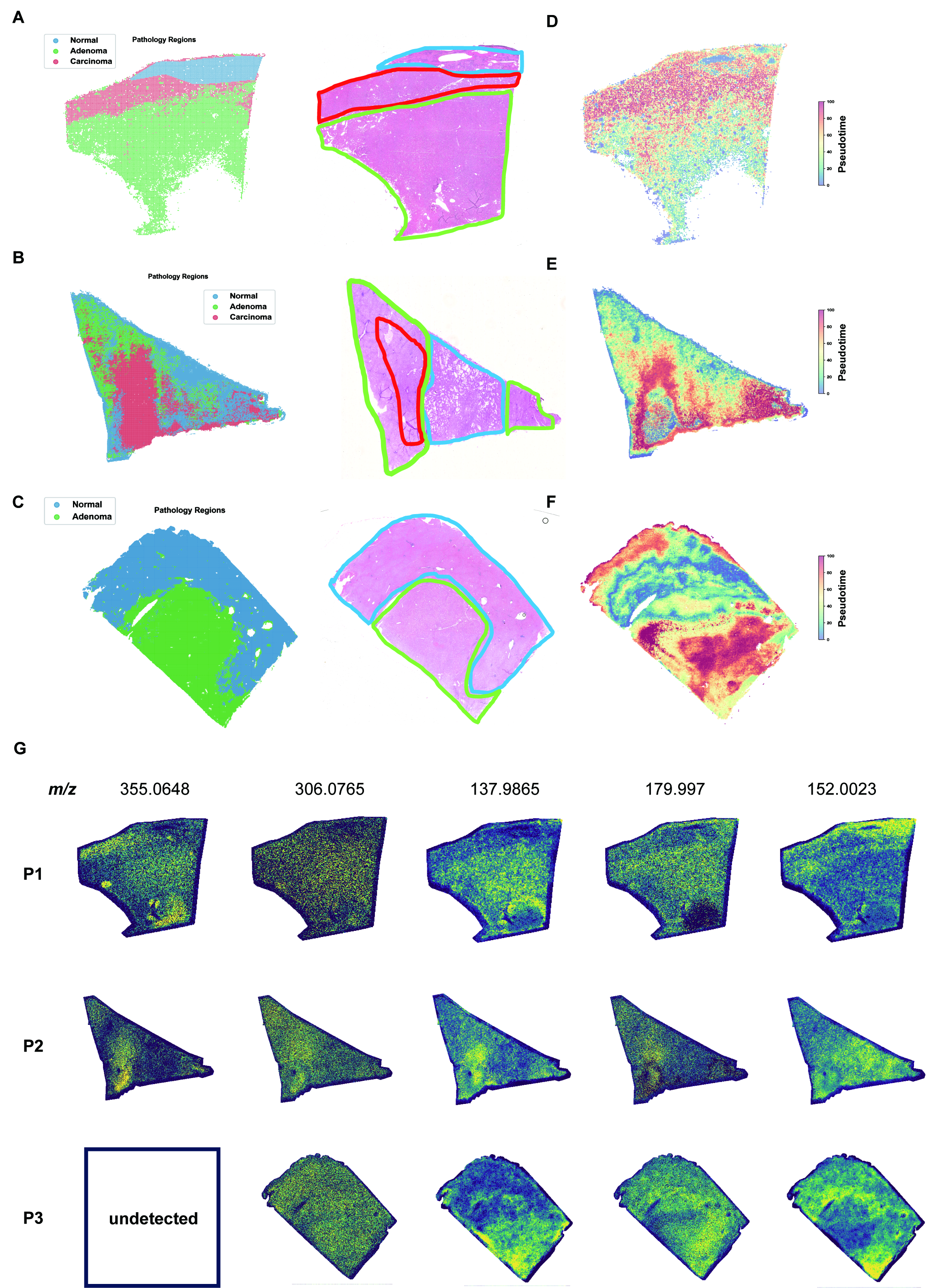

Supplement: Supplementary file 1 [file metabolites-16-00217-s001.zip › Figure S3.tif]

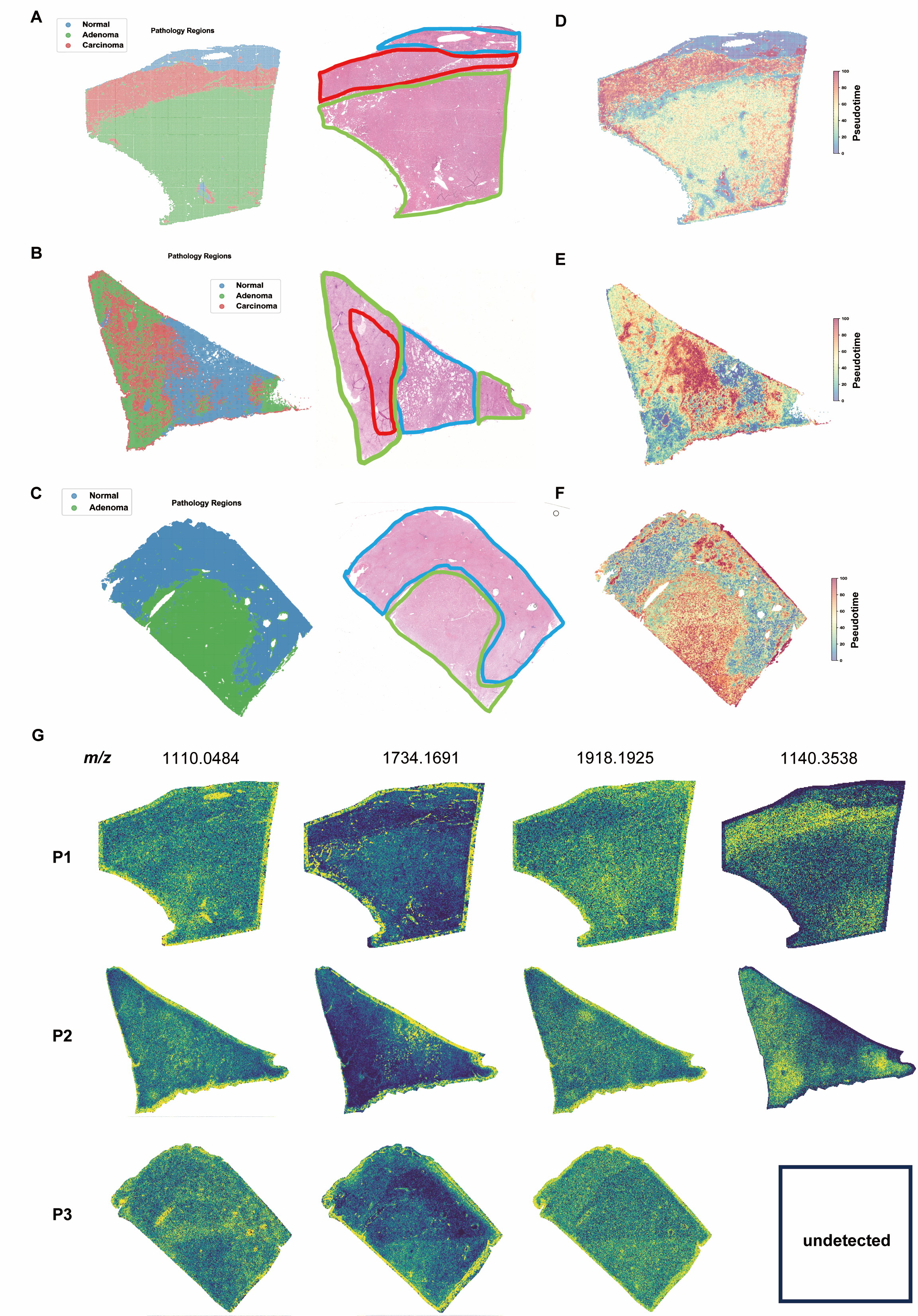

Supplement: Supplementary file 1 [file metabolites-16-00217-s001.zip › Figure S4.tif]
